# ST Segment Elevation Is Not Always Myocardial Infarction: A Case of Focal Myopericarditis

**DOI:** 10.1155/2017/3031792

**Published:** 2017-11-29

**Authors:** Cyrus M. Munguti, Samuel Akidiva, Jacob Wallace, Hussam Farhoud

**Affiliations:** ^1^Internal Medicine Department, University of Kansas Medical School, Wichita, KS, USA; ^2^Radiology Department, University of Kansas Medical School, Wichita, KS, USA; ^3^Heartland Cardiology, Wichita, KS, USA

## Abstract

Protocols exist on how to manage STEMI patients, with well-established timelines. There are times when patients present with chest pain, ST segment elevation, and biomarker elevation that are not due to coronary artery disease. These conditions usually present with normal coronary angiography. We present a case that was clinically indistinguishable from STEMI and that was diagnosed with focal myopericarditis on cardiac MRI.

## 1. Introduction

Typical chest pain with elevated ST segment elevation on electrocardiogram (EKG) is a medical emergency, with well-drilled protocols and timeline targets [1]. There are times when the ST elevation is not due to coronary obstruction but a mimic. Inflammation associated with myopericarditis can produce chest pain and EKG changes that may be clinically indistinguishable from a coronary event. Myalgia, fatigue, pleuritic chest pain, and fever are common presentations associated with viral illness, but the absence of this history does not preclude post viral myopericarditis. Typical cardiac chest pain and elevated cardiac enzymes can be present in acute coronary syndrome (ACS) as well as in myopericarditis, especially its focal form. Few case reports have been published on this rare and important mimic [2, 3]. We present a case of a young male who presented with indistinguishable features and was treated as STEMI, later to turn out as focal myocarditis.

## 2. Case Presentation

**Figure 1:**
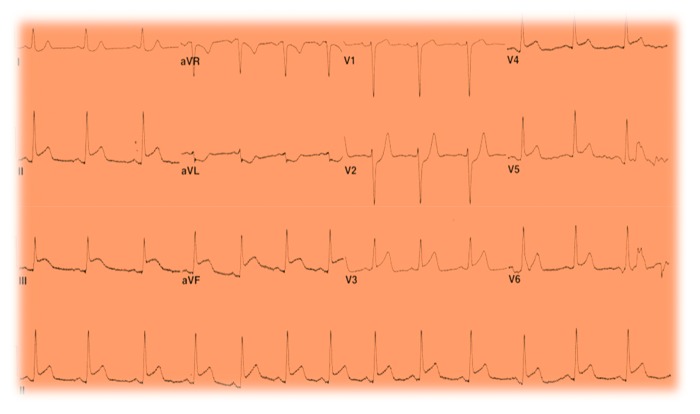
Admission EKG showing ST segment elevations in leads II, III, AVF, and V3-V5.

**Figure 2:**
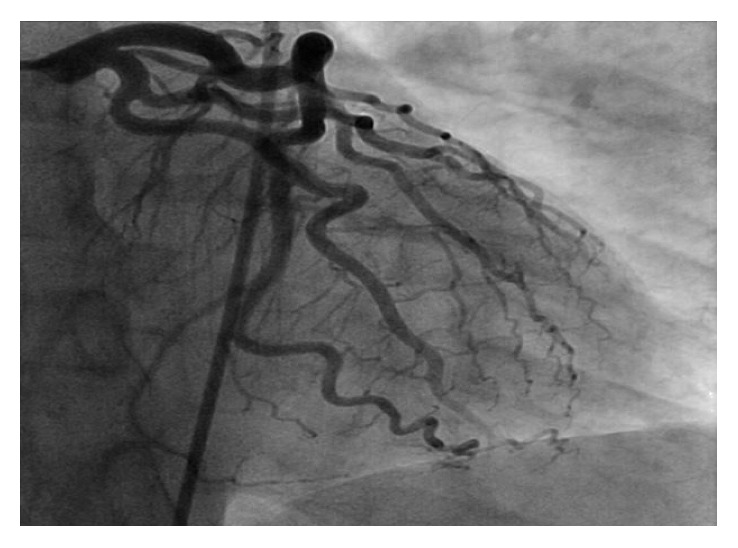
Left coronary angiogram with normal left coronary anatomy with no obstructing atheroma.

**Figure 3:**
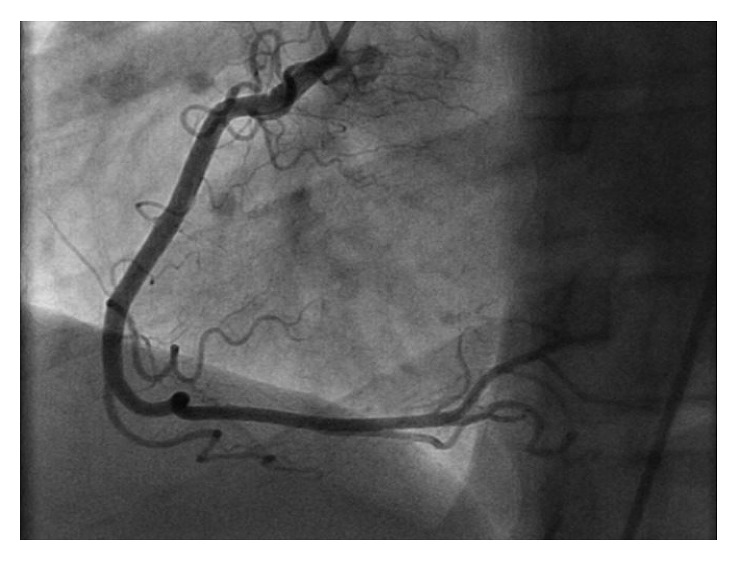
Right coronary angiogram with normal left coronary anatomy with no obstructing atheroma.

**Figure 4:**
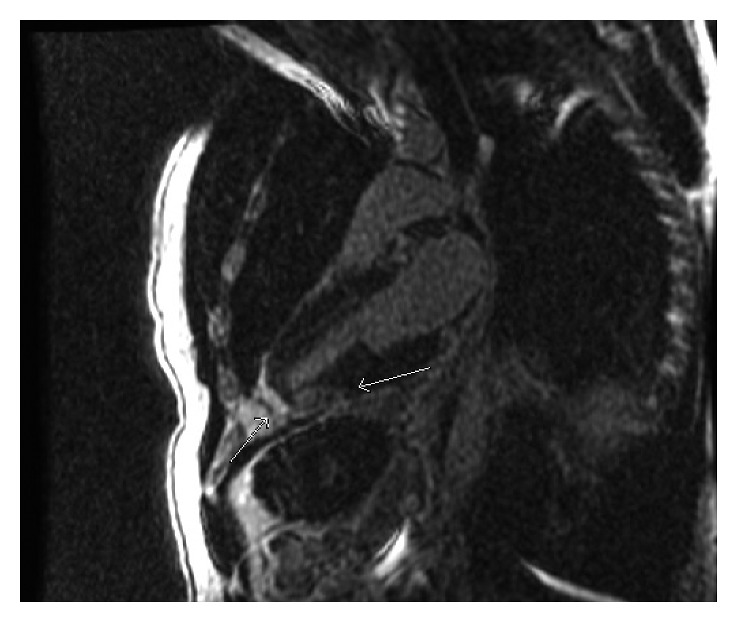
Delayed postcontrast cardiac MRI 2-chamber view demonstrates midmyocardial enhancement in the inferior wall of the left ventricle adjacent to the apex. The subendocardial region is sparred (right arrow). Mild pericardial delayed enhancement (left arrow).

**Figure 5:**
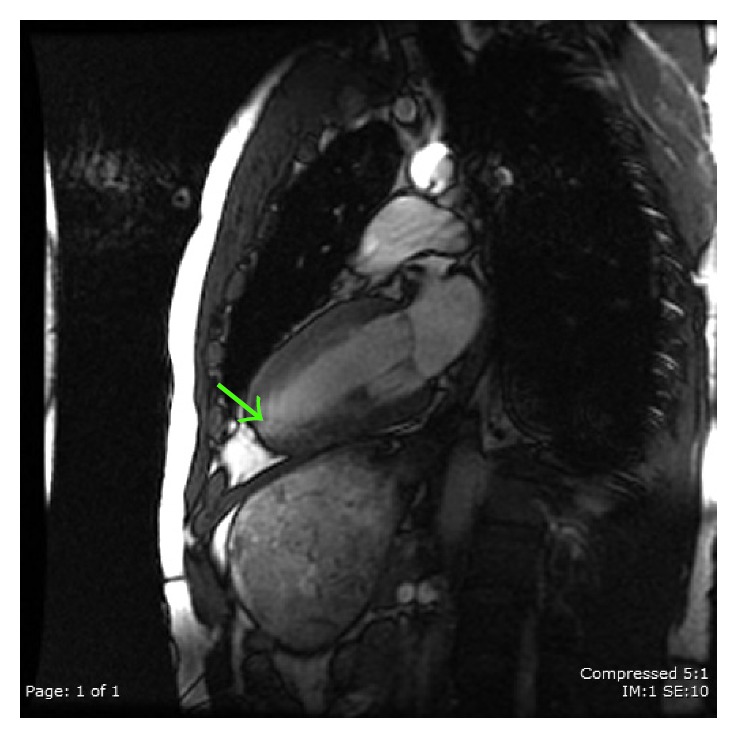
Corresponding T2 images of cardiac MRI 2-chamber view with an apical focal area of high T2 signal intensity in midmyocardium indicating focal edema at the apex.

**Figure 6:**
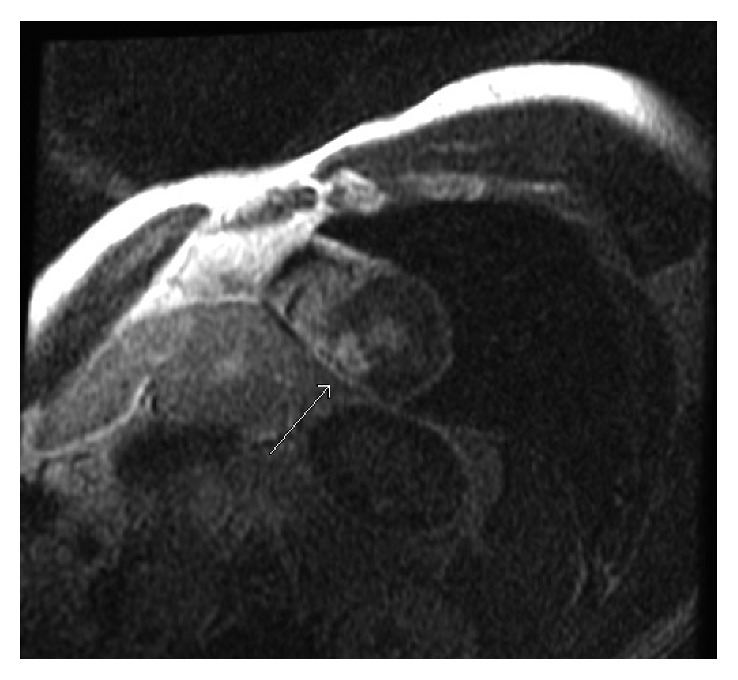
Delayed postcontrast cardiac MRI short axis view demonstrating midmyocardial enhancement of the inferior wall of the left ventricle adjacent to the apex. Subendocardial sparring is again seen.

**Figure 7:**
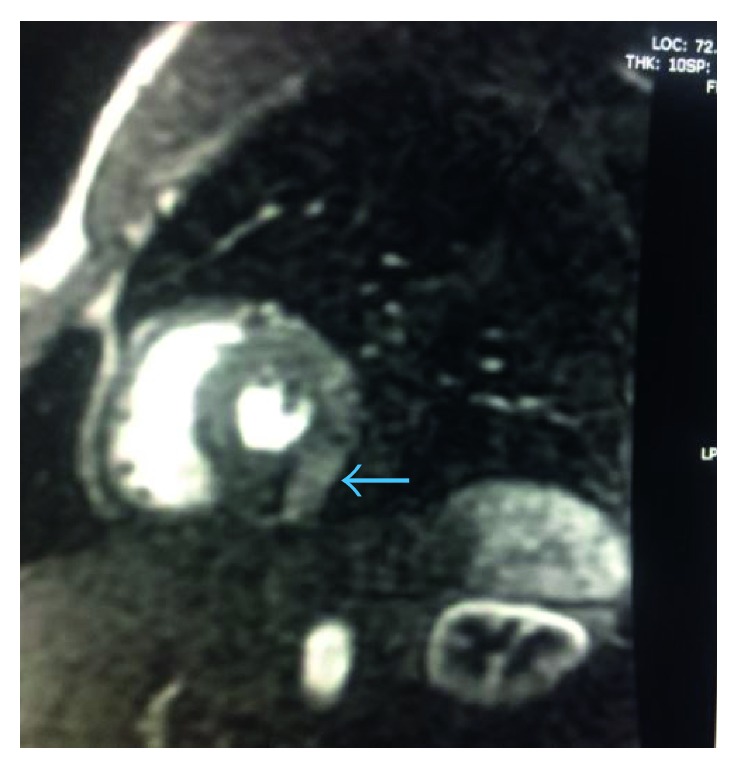
T1-weighted cardiac MRI short axis view demonstrating early enhancement ratios in the posterior epicardium and myocardium soon after gadolinium injection with sparing of the subendocardium (blue arrow).

A previously healthy 33-year-old white male presented with sudden onset substernal chest pain that started while exercising on a treadmill one hour before. He described a left sided sharp, nonradiating pain that persisted till he presented to the ER. He had associated nausea, diaphoresis, and shortness of breath. Nothing made it better. He denied heartburn, vomiting, cough, fever, and recent travel. He had no personal or family history of heart disease. On physical examination, he was a young athletic male with normal vital signs, and he appeared in distress from the pain. His cardiovascular examination was normal with no murmurs or pericardial rubs. He had an elevated troponin I at 21.9 ng/ml and an EKG ST segment elevation in the inferior leads (Figure 1). All other baseline laboratory tests were within normal limits. A STEMI alert was placed, and patients had an emergent left cardiac catheterization that reported normal coronary anatomy with no obstructing coronary stenosis (Figures 2 and 3). A left ventriculogram was also normal. He was started on a heparin drip and transferred to the coronary care unit. A plain chest X-ray did not reveal any pulmonary lesions or consolidation, and a chest CT angiogram ruled out pulmonary embolism. A transthoracic echocardiogram done reported a normal left ventricular ejection fraction (EF 50–55%) and a slight enlargement of the right ventricle without any wall motion abnormalities. Two days after presentation, the patient still reported continued chest pain and had an episode of nonsustained ventricular tachycardia (NSVT). At this point, a cardiac MRI was done (Figures 4–7) that demonstrated epicardial and midmyocardial enhancement in the inferior wall, sparing of the subendocardial region, and overlying focal pericardial enhancement, consistent with EKG changes. He was started on indomethacin; his symptoms improved in the following 5 days, and he was discharged.

## 3. Discussion

The presentation of myopericarditis is widely variable from asymptomatic to focal or diffuse myopericarditis, congestive failure, and even sudden cardiac death. Diffuse myopericarditis has well variable presentation and EKG changes, reflecting the degree of myonecrosis. Certain changes on EKG are associated with myocarditis rather than pure pericarditis, such as ST segment elevations and occurrence of arrhythmias as was evident in our patient [4]. Focal myopericarditis on the other hand may have EKG findings indistinguishable from STEMI as is seen in our case. Chest pain with unusual cardiac risk profile and normal coronary angiography should raise suspicion of focal myopericarditis. The gold standard for diagnosis of myocarditis is endomyocardial biopsy, which has variable sensitivity of up to 64% [5]. The noninvasive cardiac MRI is increasingly being used to make the diagnosis of myocarditis and is associated with sensitivity of up to 90% in one study [6, 7]. The pattern of myocarditis on MRI includes focal or global calculated myocardial early enhancement ration greater than 4.5 compared to skeletal muscles, focal or global intense T2 signal indicative of edema, or late gadolinium enhancement with nonregional ischemic distribution. These often involve epicardium towards myocardium, typically sparing the subendocardium, while myocardial infarction displays a pattern of enhancement involving the subendocardium [8–10]. Using the Lake Louise Consensus criteria [10], our patients' findings were consistent with myocarditis by displaying early gadolinium enhancement ratios, regional T2 signal edema, and myocardial late gadolinium enhancement. It is arguable that the region of cardiac MRI findings could have been consistent with a spontaneously reperfused coronary artery disease, and this theory could not be proven in our case. Our patient had no traditional risk factors for coronary artery disease, though the sudden onset of symptoms pointed to ACS and the coronary angiograms was clear of atherosclerosis.

Similar presentations to this patient that were ruled out include Takotsubo cardiomyopathy as the ventriculogram was normal during cardiac catheterization. The coronary anatomy was normal, and thus both coronary artery disease and spontaneous coronary dissection were ruled out. Our patient had no eosinophilia on peripheral blood testing; hence, hyper eosinophilic myocarditis and hypersensitivity myocarditis were also ruled out.

## 4. Conclusion

Focal myopericarditis may present with typical chest pain, ST segment elevation on EKG, and biomarker elevation that may be indistinguishable from STEMI. Unusual cardiac risk profile, in absence of traditional cardiac risk factors, should raise suspicion of alternative diagnosis. Cardiac MRI is useful in distinguishing myopericarditis from MI.
